# Cavitary nodule caused by *Emergomyces orientalis* in a diabetic patient: a case report

**DOI:** 10.3389/fmed.2026.1829356

**Published:** 2026-04-29

**Authors:** Yajie Zhou, Luying Chen, Lixia Wang, Zhuoyang Zhao, Junwei Tu, Huijun Chen, Saibin Wang

**Affiliations:** 1 School of Medicine, Shaoxing University, Shaoxing, Zhejiang, China; 2 Department of Pulmonary and Critical Care Medicine, Nanhu District People’s Hospital of Jiaxing City, Jiaxing, Zhejiang, China; 3 Department of Pathology, Jinhua Municipal Central Hospital, Jinhua, Zhejiang, China; 4 Department of Pulmonary and Critical Care Medicine, Jinhua Municipal Central Hospital, Jinhua, Zhejiang, China

**Keywords:** cavitary nodule, *Emergomyces orientalis* emergomycosis, fungal diagnosis, metagenomic next-generation sequencing, precision medicine

## Abstract

*Emergomyces orientalis* is a rare thermally dimorphic fungus belonging to the family *Ajellomycetaceae*. It exists in the environment as a mold producing conidia, which are inhaled and transform into yeast-like cells at body temperature to cause disseminated infections. While primarily associated with immunocompromised individuals, especially those with HIV. Diagnosis remains challenging due to its morphological similarity to Blastomyces dermatitidis and the frequent failure of routine cultures. Thus, molecular methods such as metagenomic next-generation sequencing (mNGS) have become crucial for early identification. This case report describes a 51-year-old man with type 2 diabetes mellitus presented (T2DM) with a 10-day history of back pain, pharyngeal discomfort, and scant sputum. Chest CT showed multiple bilateral pulmonary nodules, one of which had cavitated. mNGS of a percutaneous lung biopsy confirmed *Emergomyces orientalis*. Histopathology also supported the diagnosis. The patient was discharged on oral itraconazole after partial symptomatic improvement, with outpatient follow-up arranged. Two months of antifungal therapy resulted in mild reduction of cavitary lesions on follow-up CT.

## Introduction

*Emergomyces orientalis*, a thermally dimorphic fungus causes disseminated emergomycosis, primarily affecting immunocompromised hosts (1). This species, endemic to Asia, contrasts with its phylogenetic relatives: *E. africanus* in Southern Africa and *E. canadensis* in North America (2). Although a systematic review indicates that Human Immunodeficiency Virus (HIV) coinfection accounts for 79.2% of global emergomycosis cases (3), predominantly in individuals with advanced immunosuppression, recent reports describe a growing number of infections in non-HIV populations, including transplant recipients and patients receiving immunosuppressive therapies. *E. orientalis* demonstrates tissue-invasive capability and induces chronic granulomatous inflammation in host tissues (1). We report a rare case of *E. orientalis* emergomycosis in a diabetic patient whose only immunocompromising condition was type 2 diabetes mellitus (T2DM), without HIV or immunosuppressive therapy. A conventional chest CT revealed cavitary pulmonary nodule. Although cavitary lesion has been observed in lung tissue on pathological examination (4), to our knowledge, this represents their first detailed description on conventional chest CT imaging. Diagnosis relied on mNGS of lung tissue, bypassing limitations of conventional culture methods.

## Case presentation

A 51-year-old male with T2DM presented with a 10-day history of dorsalgia accompanied by pharyngeal discomfort, chest tightness, and scant sputum production. The scant sputum was not unusually discolored and had no identifiable aggravating or alleviating factors. Furthermore, the patient reported no fever, hemoptysis, or weight loss. Initial conventional chest CT at a peripheral facility revealed multiple pulmonary nodules in both lungs, with one lesion showing cavitation. Upon admission, the patient denied any history of HIV, corticosteroid use, or malignancy.

Physical examination revealed stable hemodynamics: blood pressure was 140/84 mmHg [reference: < 130/80], heart rate was 118 beats per minute and regular [reference: 60–100], respiratory rate was 20 breaths per minute, and he was afebrile (temperature 36.1 °C). Physical examination revealed that the lungs were clear to auscultation bilaterally. Laboratory investigations showed preserved hematological parameters [leukocytes, 6.09 × 10^9^/L (reference range: 3.50–9.50)] and a normal C-reactive protein level [< 0.05 mg/dL (reference range: < 8.00)]. Serum biochemistry was unremarkable except for elevated hemoglobin A1c [8.30% (reference range: 4.60–6.50)] and dyslipidemia, with total cholesterol of 6.28 mmol/L (reference range: 3.10–5.95) and low-density lipoprotein of 4.29 mmol/L (reference range: < 3.37). Mycological workup was non-diagnostic, with a (1,3)-β-D-glucan level of < 37.50 pg./mL (reference range: < 70.00), assayed using a Gram-negative bacterial lipopolysaccharide detection kit [Dana (Tianjin) Biotech Co., China] on a fully automated enzyme immunoassay analyzer; Galactomannan antigen: negative, determined by a manual enzyme-linked immunosorbent assay (ELISA) without the use of an automated instrument. Besides, three sputum cultures were performed, all of which grew normal respiratory flora with no significant pathogens isolated. Tuberculosis screening (T-SPOT, acid-fast bacilli smears negative) was likewise negative. Additionally, the patient’s HIV serology was negative.

**Figure 1:**
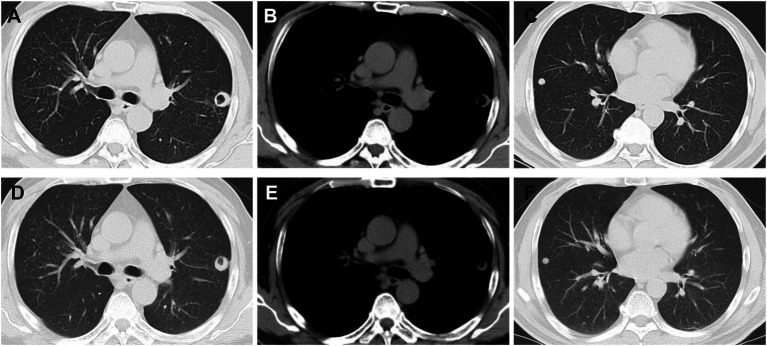
**(A–C)** CT shows miliary nodular opacities with cavitary evolution in one lesion (Series 301/Image 26), approximately 16 × 15 mm. **(D–F)** CT shows miliary nodular opacities with cavitary evolution in one lesion (Series 201/Image 25), approximately 16 × 14 mm.

Follow-up conventional chest CT demonstrated diffuse bilateral miliary nodular opacities with cavitary evolution in one lesion (Series 301/Image 26), approximately 16*15 mm, concurrent with multistation mediastinal and bilateral hilar lymphadenopathy exhibiting mild enlargement (Figures 1A–C). Given the atypical radiographic manifestations, empirical antimicrobial therapy with intravenous piperacillin-tazobactam (4.5 g q8h) was administered for 3 days to cover potential atypical respiratory pathogens. This regimen was selected to cover fastidious Gram-negative bacilli, including pathogens such as *Haemophilus influenzae* and *Klebsiella pneumoniae*. Additionally, it provides coverage against anaerobic bacteria. *Pseudomonas aeruginosa* was also considered a potential pathogen, particularly given the risk in patients with underlying immunocompromised status.

**Figure 2:**
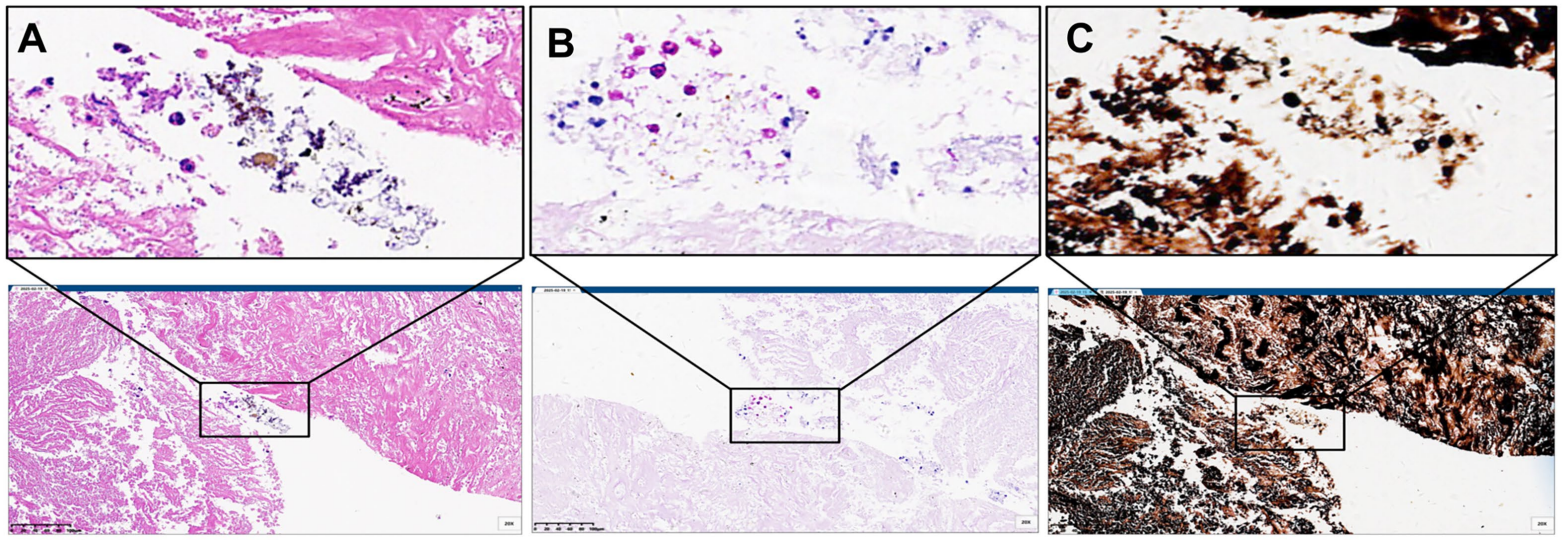
**(A)** The hematoxylin and eosin staining patterns indicate possible mycosis in the lung tissue. **(B)** The periodic acid–Schiff (PAS) staining patterns indicate possible mycosis in the lung tissue. **(C)** The Grocott’s methenamine silver (GMS) stain indicate possible mycosis in the lung tissue.

Given the indeterminate diagnostic status, bronchoscopic bronchoalveolar lavage (BAL) with targeted next-generation sequencing (tNGS). The tNGS was conducted on the GridION sequencer (Oxford Nanopore Technologies, UK) using an in-house kit, which revealed no pathogenic signatures. Cytopathological examination identified scant necrotic cellular debris without definitive malignant or infectious features. Then, CT-guided percutaneous lung biopsy were performed, mNGS of the lung biopsy specimen was performed on the BGI sequencing platform with an in-house kit, which detected *E. orientalis* with high specificity (14,771 unique sequence reads), confirming the disseminated fungal infection. Histopathological evaluation demonstrated characteristic spores exhibiting positive periodic acid–Schiff (PAS) and Grocott’s methenamine silver (GMS) stain (Figures 2A–C). The patient was discharged on a regimen of oral itraconazole (200 mg once daily, administered as two 100 mg capsules after meals). The decision for discharge was based on his subjective clinical improvement, particularly the alleviation of back pain, and the confirmed microbiological diagnosis. Since the inflammatory markers were within normal limits at admission and significant radiological changes were not anticipated over such a short period, conventional chest CT was not performed during the brief hospital stay. He was instructed to return for an outpatient follow-up in two weeks to assess treatment efficacy and monitor for potential adverse effects of itraconazole, including liver function tests.

Following discharge, the patient adhered to the prescribed medication regimen. At the one-month outpatient follow-up, liver and renal function tests were within normal limits, and a conventional chest CT scan showed findings stable compared to the previous study. The same antifungal regimen was therefore continued. A follow-up conventional chest CT two months later demonstrated a reduction in the size of the cavitary lesion (Figures 1D–F). Subsequently, the patient was unable to return for the final imaging reassessment due to relocation, precluding objective radiological confirmation of complete lesion resolution. However, telephone follow-up revealed that the patient reported substantial symptomatic improvement with no residual impact on daily activities.

## Discussion and conclusion

The pathogenesis of emergomycosis begins with the inhalation of environmental conidia, which then transition into yeast-like forms within the host. Then the disseminated mycosis characterized by hematogenous spread to multiple organ systems including cutaneous, pulmonary, and osseous tissues (5). *E. orientalis* exhibits potential divergence in pathogenic mechanisms compared to other *Emergomyces* species. Current understanding of its molecular pathogenesis remains incomplete, necessitating systematic investigation into its unique molecular signatures and specific modes of host-pathogen interaction. The disease carries a grave prognosis, particularly among patients with advanced HIV. In a South African cohort of patients with disseminated emergomycosis (predominantly HIV-positive), the all-cause mortality was 48%, with an infection-attributable mortality of 51%, despite the administration of amphotericin B-based therapy (6). This underscores the critical need for improved diagnostic and therapeutic strategies.

The cavitary nodule identified in our patient constitutes a novel imaging hallmark of *E. orientalis* infection, distinguishing it from previously documented non-cavitary presentations (miliary nodules or consolidations) in four Asian cases reported by Wang et al. (4, 7–9) (Table 1). Notably, one of these cases described a male patient with T2DM and no other immunodeficiencies whose conventional chest CT revealed multiple nodular densities in the left lower lung—distinctly different from the imaging findings in our reported patient. The imaging findings of cavitary pulmonary nodule in this patient require careful differential diagnosis, particularly in a region with a high prevalence of tuberculosis and in a patient with underlying T2DM. First, pulmonary tuberculosis remains a primary consideration, although negative T-SPOT. TB assay and acid-fast bacilli smears reduce its likelihood. Second, bacterial lung abscesses typically present as thick-walled cavities with air-fluid levels and are associated with a more acute, toxic clinical presentation, which is inconsistent with the findings in this case. Third, among endemic dimorphic fungi, Histoplasma capsulatum can manifest identically. Finally, non-infectious etiologies such as granulomatosis with polyangiitis or squamous cell carcinoma must also be considered. We hypothesize that the mechanism underlying the cavitary nodule in this patient may parallel in chronic necrotizing aspergillosis: diabetes-associated microangiopathy may exacerbate tissue necrosis following fungal angioinvasion (10). However, the precise mechanism requires elucidation in future studies. Doctors should recognize that cavitation does not exclude rare fungal etiologies, necessitating mNGS confirmation when conventional cultures yield negative results in endemic regions.

**Table 1:** Summary of major cases of *Emergomyces orientalis* reported in the literatures.

Case	Year	Region/nation	Age/sex	Medical history	Clinical manifestations	Diagnostic samples and method	Imaging findings	Treatment
1	2017	Shanxi, China	64/male	T2DM	Productive cough, fever, pustular lesions on neck, subcutaneous nodules	Sputum/Sputum fungal culture	multiple nodular densities in the left lower lung	Amphotericin B deoxycholate plus fluconazole, followed by itraconazole
2	2021	Tibet, China	41/male	Kidney transplant recipient	Progressive right lower chest pain, mild cough with sputum	BALF/mNGS	Pulmonary consolidation with air bronchograms	Oral itraconazole, decreased tacrolimus dosage, later added flucytosine and posaconazole
3	2024	Aba Prefecture, China	52/ male	SLE	Cough, phlegm, fever, headache, nausea	Bone marrow/mNGS	micronodule in the middle lobe of the right lung, scattered interstitial changes	Intravenous amphotericin B cholesterol sulfate complex, later oral itraconazole
4	2024	Hebei, China	52/male	Coal mining work history	Intermittent fever, night sweats, coughing and phlegm	BALF/mNGS	Chest CT revealed multiple soft-tissue nodules in both lungs	Amphotericin B cholesterol sulfate complex injection, later oral voriconazole

T2DM, Type 2 diabetes mellitus; SLE, systemic lupus erythematosus; BALF, bronchoalveolar lavage fluid; CT, computed tomography; mNGS, metagenomic next-generation sequencing.

The reasons for the discordance between the negative tNGS result from BAL fluid and the positive mNGS result from tissue biopsy are as follows. First, due to the anatomical location and sampling technique, the lesion in our case was situated in the lung periphery, distal to the hilum and near the pleura. This location prevented the bronchoscope from advancing deep enough, so the lavage fluid could hardly reach the core of the lesion. Moreover, flushing with normal saline diluted the pathogen concentration below the detection threshold of mNGS. In addition, targeted NGS (tNGS) is limited by its predefined panel, making it difficult to detect unknown or low-abundance pathogens. In contrast, the second sampling directly obtained lung tissue from the lesion, overcoming the dilution issue and capturing high-density pathogen DNA (including from intracellular bacteria, fungi, biofilms, etc.), thereby greatly improving the diagnostic yield. This success also benefited from the unbiased, sequence-independent nature of mNGS, which indiscriminately sequences all nucleic acids (DNA or RNA) present in a sample.

Retrospective analysis of existing case series, including our patient, reveals persistent diagnostic challenges in emergomycosis. The culture and histopathology, remain constrained by low sensitivity due to fastidious growth requirements and morphological mimicry of other dimorphic fungi. mNGS enables unbiased pathogen detection by capturing all nucleic acids in clinical samples, rapidly identifying genomic sequences to overcome culture limitations (11). For example, in immunocompromised hosts, mNGS demonstrates 92.3% sensitivity for rare fungi, significantly outperforming conventional staining methods (9.3%) (12). Although mNGS has limitations including high costs and a relatively long turnaround time, it remains a sensitive and unbiased diagnostic method. Its broad-spectrum capability demonstrates clinically valuable utility in detecting diverse infectious pathogens, providing clinicians with a complementary tool to conventional diagnostic approaches (13).

For the treatment of *E. orientalis* infection, no established therapeutic guidelines currently exist. Based on empirical experience from previous cases, the patient was empirically administered itraconazole as an antifungal regimen. The patient has achieved a preliminary symptomatic relief. However, post-discharge outcomes remain unclear, including whether symptoms resolved, whether cavitary nodule resolved on imaging, or whether potential drug-induced hepatotoxicity occurred (14). This constitutes a key limitation of this case report. Therefore, establishing evidence-based standardized treatment guidelines for this pathogen is needed to optimize clinical management.

Our case indicates that *E. orientalis* can cause disease in patients without classic immunosuppression (e.g., HIV or immunosuppressive therapy), presenting without severe symptoms such as fever and with relatively unremarkable routine laboratory parameters. Moreover, the patient’s conventional chest CT revealed cavitary nodule, a finding not previously reported for this infection. It should be noted that this study has limitations, including the lack of detailed immunological profiling and antifungal susceptibility testing (AFST), as well as the inability to conduct long-term follow-up after the patient was lost to contact after changing his residence. Despite these limitations, our findings suggest that *E. orientalis* infection is a rare disseminated mycosis with diverse manifestations. Conventional diagnostic methods exhibit limited sensitivity, whereas mNGS offers a sensitive and unbiased approach. Future research should focus on elucidating specific molecular mechanisms, optimizing diagnostic strategies, and exploring more effective antifungal regimens informed by AFST to improve clinical outcomes.

## Data Availability

The datasets presented in this study can be found in online repositories. The names of the repository/repositories and accession number(s) can be found in the article/supplementary material.
